# CAN ACUTE MUSCLE DAMAGE LEAD TO OSTEOARTHRITIS?

**DOI:** 10.1093/geroni/igad104.2481

**Published:** 2023-12-21

**Authors:** Kamal Awad, Logan Moore, Jian Huang, Leticia Brotto, Marco Brotto

**Affiliations:** University of Texas at Arlington, Arlington, Texas, United States; University of Texas Arlington, Arlington, Texas, United States; University of Texas At Arlington, Arlington, Texas, United States; University of Texas Arlington, Arlington, Texas, United States; The University of Texas at Arlington, Arlington, Texas, United States

## Abstract

Skeletal muscles are crucial for maintaining musculoskeletal homeostasis, as they provide mechanical support, joint stability, and biochemical functions via myokine secretion. We investigated whether acute muscle damage or weakness could contribute to the development of osteoarthritis (OA). Using a chemical damage model, we induced muscle damage by injecting 1.2% barium chloride into the tibialis anterior muscle, resulting in an immune response characterized by a significant increase in the concentration of Prostaglandin E2 (PGE2) and other lipid mediators derived from the EPA and DHA signaling pathways, as quantified using our innovative lipidomics methods. The muscle damage was confirmed and visualized by muscle histomorphometry. The damage resulted in muscle weakness, detected by a significant decrease in grip strength at 4-days post-injury, but without changes in mice activity measured with force-plate actimeter. After 1-month post-injury, grip strength and muscle histology returned to normal, but histological changes were observed in the articulating cartilage, indicating surface erosion and cartilage matrix loss (OARSI grade 4). We used Raman Spectroscopy and micro-CT to confirm the joint alterations observed in the changes in subchondral bone and increased bone volume fraction in the cortical shell of the subchondral bone, respectively, features characteristic of OA in rodents and humans. These findings suggest that muscle weakness or imbalance due to injury can contribute to joint degeneration. This study highlights the significance of muscles in preventing OA and provides a novel approach for exploring the relationship between muscle damage and OA development. This new model could prove valuable for aging research.

